# Mutant Huntingtin Gene-Dose Impacts on Aggregate Deposition, DARPP32 Expression and Neuroinflammation in HdhQ150 Mice

**DOI:** 10.1371/journal.pone.0075108

**Published:** 2013-09-23

**Authors:** Douglas Young, Franziska Mayer, Nella Vidotto, Tatjana Schweizer, Ramon Berth, Dorothee Abramowski, Derya R. Shimshek, P. Herman van der Putten, Peter Schmid

**Affiliations:** Novartis Institutes for BioMedical Research, Neuroscience, Novartis Pharma AG, Basel, Switzerland; Ruhr University Bochum, Germany

## Abstract

Huntington's disease (HD) is an autosomal dominant, progressive and fatal neurological disorder caused by an expansion of CAG repeats in exon-1 of the huntingtin gene. The encoded poly-glutamine stretch renders mutant huntingtin prone to aggregation. HdhQ150 mice genocopy a pathogenic repeat (∼150 CAGs) in the endogenous mouse huntingtin gene and model predominantly pre-manifest HD. Treating early is likely important to prevent or delay HD, and HdhQ150 mice may be useful to assess therapeutic strategies targeting pre-manifest HD. This requires appropriate markers and here we demonstrate, that pre-symptomatic HdhQ150 mice show several dramatic mutant huntingtin gene-dose dependent pathological changes including: (i) an increase of neuronal intra-nuclear inclusions (NIIs) in brain, (ii) an increase of extra-nuclear aggregates in dentate gyrus, (iii) a decrease of DARPP32 protein and (iv) an increase in glial markers of neuroinflammation, which curiously did not correlate with local neuronal mutant huntingtin inclusion-burden. HdhQ150 mice developed NIIs also in all retinal neuron cell-types, demonstrating that retinal NIIs are not specific to human exon-1 R6 HD mouse models. Taken together, the striking and robust mutant huntingtin gene-dose related changes in aggregate-load, DARPP32 levels and glial activation markers should greatly facilitate future testing of therapeutic strategies in the HdhQ150 HD mouse model.

## Introduction

Huntington's disease (HD) is a progressive fatal neurodegenerative disease caused by CAG repeat expansions in the *huntingtin* (*HTT*) gene. The repeat encodes a stretch of glutamines (polyQ) near the N-terminus of huntingtin (HTT). Recent findings suggest that cleavage of full-length mutant huntingtin into smaller N-terminal fragments is a key process in the pathophysiology of HD [1], [2], [3]. N-terminal fragments are also encoded by a small exon-1 mRNA that is generated by miss-splicing [4]. The polyQ renders these fragments prone to form soluble aggregates, large insoluble extra-nuclear aggregates and neuronal intra-nuclear inclusions (NIIs). These neuropathological hallmarks of HD [5], [6], [7] were thought to cause neuronal toxicity [8] but evidence has mounted, that aggregate formation may provide neuroprotection by neutralizing toxic soluble mutant huntingtin fragments [9], [10], [11].

Several genetic mouse models have been developed to mimic and investigate the neuropathophysiological processes involved in HD [12]. The transgenic human exon-1 R6/2 mouse HD model [13] has been mainstay for the preclinical testing of therapeutic strategies because these mice show an early-onset and rapid progression of disease accompanied by an accumulation of mutant huntingtin aggregates. The R6/2 mouse less precisely genocopies HD as compared to knock-in lines, which express full-length mutant huntingtin [14], [15]. Knock-in mice develop phenotypes and neuropathology late in life, they have not been used widely to test therapeutics, and they seem to model mainly pre-manifest HD. However, since starting treatments early is likely important to prevent or delay HD, there is a need to advance methods for detecting human (mHTT) and mouse (mHtt) mutant huntingtin [16], [17], and markers for early and progressive pathogenic changes in the brain of full-length HD models during the pre-manifest stage of disease.

Full-length HdhQ150 mice carry ∼150 CAG repeats in their mutant endogenous mouse gene (*mHtt)*. Such a repeat-size would cause childhood-onset HD. By two years of age, HdhQ150 mice have a widespread brain and peripheral aggregate pathology [18], [19], [20] that is similar to R6/2 mice [14]. Behavioural abnormalities start late by 22 months of age [14] and like other full-length models [21], also HdhQ150 mice manifest some early mHtt-related changes long before behavioural symptoms appear [19], [22]. Provided such changes are progressive, sensitive and quantifiable, they might serve as surrogate markers to assess pre-clinically therapeutic strategies for early treatment in HD. Baldo et al. [16] recently described quantitative and qualitative changes in mHtt protein species. These authors demonstrated that HdhQ150 mice contain a soluble pool of mHtt fragments (oligomers). This pool is present already shortly after birth, it declines with age and its size inversely correlated with an increased load of insoluble aggregates. It has also been noted that NII formation in HdhQ150 mice increased in mice carrying two copies of the *mHtt* gene as compared to those carrying only one copy [17].

In this report we investigated, to what extent *mHtt* gene-dose determines aggregate accumulation together with alterations in other brain markers of pathogenic changes. In humans, two *mHTT* gene copies correlate with a more severe clinical disease course [23]. Increased CAG repeat-length, which may also influence mutant huntingtin levels by enhancing translation [24], is inversely correlated with age of onset [25], [26]. In mice, higher transgene levels and *mHtt* gene-dose affect both disease onset and disease progression [13], [27], [28], [29], and siRNA-mediated downregulation of huntingtin attenuates neuropathology and behavioral deficits [30], [31], [32], [33] Altogether, these findings suggest that markers that change as a function of *mHtt* gene-dose are physiologically highly relevant. Here we demonstrate, that doubling *mHtt* gene-dose in HdhQ150 mice dramatically accelerated, in a quantifiable fashion, the formation of intra-nuclear mHtt deposits (named neuronal intra-nuclear inclusions or NIIs hereafter) and of extra-nuclear mHtt deposits (named aggregates hereafter). It also exacerbated the reduction in DARPP32 seen in mice carrying a single *mHtt* allele, and it increased the expression of glial neuroinflammatory markers. Altogether, the monitoring of these markers should greatly facilitate the assessment of mHtt-lowering strategies in the HdhQ150 HD mouse model.

## Materials and Methods

### Ethics statement

All animal experiments were carried out in accordance with authorization guidelines of the Swiss Federal and Cantonal veterinary offices for the care and use of laboratory animals. Studies described in this report were approved by the Swiss Cantonal veterinary offices (Basel-City) and performed according to Novartis animal license numbers 2063, 2382, 2415 and 1858.

### Mice

The HdhQ150 C57BL/6 mouse line was obtained from Prof. Gillian Bates (King's College London) in agreement with Dr. Peter Detloff and the University of Alabama at Birmingham Research Foundation. HdhQ150 mice were maintained on a C57BL/6J background. Heterozygous HdhQ150 mice (named HdhQ150 HET hereafter) were crossed with C57BL/6J wildtype mice to obtain transgenic and wildtype siblings. Homozygous HdhQ150 mice (named HdhQ150 HOM hereafter) were generated by breeding heterozygous HdhQ150 males and females. The R6/2 mouse line was obtained from Prof. Gillian Bates (King's College London) and bred and maintained on a mixed C57BL/6J×CBA/Ca background by mating transgenic R6/2 males and C57BL/6J×CBA/Ca F1 females to obtain transgenic and wildtype siblings.

### Genotyping and determining CAG repeat-length

To confirm that CAG repeat-lengths in HdhQ150 HET and HdhQ150 HOM mice were maintained at comparable lengths, we performed genotyping of HdhQ150 mice by PCR (oligonucleotides used: oIMR3355 (5′-cccattcattgccttggctg-3′) and oIMR3356 (5′-gcggctgagggggttga-3′)) using expand High Fidelity PCR System (Roche); final concentrations: 0.2 mM dNTPs, 2 µM of each oligonucleotide, 1.8 M betaine, total volume 25 µl; thermal profile: 94°C 5 min; 10 cycles 94°C 30 sec, 53°C 30 sec, 72°C 1 min; 20 cycles 94°C 30 sec, 53°C 30 sec, 72°C 1 min (plus an additional cycle elongation of 5 sec for each subsequent cycle); 72°C 7 min). DNA was obtained from tails or ear biopsies using DirectPCR Lysis Reagent (peqlab). Genotyping of R6/2 was performed by PCR as follows: oligonucleotides 40256-FWD (5′-gagtccctcaagtccttccagca-3′) and 40261-RVS (5′-gcccaaactcacggtcggt-3′); GC-RICH PCR System (Roche): 0.2 mM dNTPs, 0.2 µM of each oligonucleotide, 0.2 M betaine, total volume 25 µl; thermal profile: 95°C 3 min; 10 cycles 95°C 30 sec, 62°C 30 sec, 72°C 45 sec; 25 cycles 95°C 30 sec, 62°C 30 sec, 72°C 45 sec (+ cycle elongation 5 sec for each cycle in addition); 72°C 7 min). PCR products were analysed and CAG repeat-lengths were determined using a Qiaxcel column-electrophoreses apparatus (Qiagen). DNA was obtained from tail or ear biopsies lysed with DirectPCR Lysis Reagent (peqlab). The CAG repeat lengths of HdhQ150 mice were between 130 and 170.

### Antibodies for immunohistochemistry

MW8 (dilution 1∶1000): a monoclonal antibody developed by Paul Patterson [34], [35] was obtained from the Developmental Studies Hybridoma Bank developed under the auspices of the NICHD and maintained by The University of Iowa, Department of Biological Sciences, Iowa City, IA 52242. MW8 labelled with fluorescence dyes (*Alexa, Invitrogen)* was used for double staining experiments. Anti-DARPP32 (dilution 1∶500; clone 19A3, monoclonal rabbit IgG; Cell Signaling) reacts with dopamine- and cyclic AMP-regulated phosphoprotein, 32 kDa, a cytosolic protein that is highly enriched in striatal medium spiny neurons. Anti-Iba1 (dilution 1∶200; polyclonal rabbit antiserum; WAKO chemicals, #019-19471) reacts with ionized calcium-binding adaptor molecule 1 (Iba1), a calcium binding EF hand protein, which is specifically expressed in microglia and macrophages. Anti-GFAP (dilution 1∶5000) polyclonal rabbit antiserum (DAKO #Z0334), binds to a 50 kDa intra-cytoplasmic filamentous protein that is part of the cytoskeleton in astrocytes. The antibody also strongly reacts with activated Müller glia cells in the retina. Secondary Antibodies (dilution 1∶500) used include: Alexa 488-labeled or Alexa 594-labeled goat anti-rabbit IgG (Invitrogen); Alexa 488-labeled or Alexa 594-labeled goat anti-mouse IgG (Invitrogen).

### Automated immunohistochemistry of paraffin sections

4 µm para-sagittal paraffin sections were mounted on SuperFrost+ slides and automatically immunostained using the Discovery XT technology (Ventana/Roche diagnostics). Briefly, sections were deparaffinized, rehydrated, subjected to antigen retrieval by heating with CC1 cell conditioning buffer (Ventana/Roche Diagnostics), incubated for 60 min at room temperature with primary antibody diluted in antibody diluent (Ventana/Roche Diagnostics), incubated with the respective biotinylated secondary antibody diluted in Ventana antibody dilution, reacted with DABMab kit (Ventana/Roche Diagnostics) and counterstained with blueing reagent (Ventana/Roche Diagnostics).

### Manual immunofluorescence staining of paraffin sections

4 µm sagittal paraffin sections were de-waxed and subjected to antigen retrieval by microwaving for 10 min at 98°C in 0.1 M citrate buffer pH 6.0 and rinsed in PBS. Sections were subsequently incubated for 1 h with PBS containing 2% goat serum, reacted over night at 4°C with antibody diluted in PBS containing 2% goat serum, washed 3 times in PBS, incubated for 1 h at room temperature with Alexa594-labeled and/or Alexa488-labeled secondary antibody (Invitrogen) diluted in PBS, and mounted with Prolong Gold containing DAPI nucleic acid counterstain (Invitrogen).

### Manual immunofluorescence staining of frozen sections

10 µm frozen sagittal sections were prepared from snap frozen brain hemispheres, placed onto SuperFrost+ glas slides and air dried for 10 min. Sections were then fixed for 30 min at room temperature with 4% paraformaldehyde in PBS pH 7.4, rinsed 3 times for 5 min in PBS, incubated for 1 h with PBS containing 2% goat serum, reacted overnight at 4°C with antibody diluted in PBS containing 2% goat serum, washed 3 times in PBS, incubated for 1 h at room temperature with Alexa594-labeled and/or Alexa488-labeled secondary antibody (Invitrogen) diluted in PBS, and mounted with Prolong Gold containing DAPI nucleic acid counterstain (Invitrogen).

### Digital image analysis and statistical evaluation of DARPP32 immunostainings

Digital slides were generated with a slide scanner (Mirax/Zeiss) equipped with an Axiocam camera (Zeiss) for fluorescence images, and a Marlin camera (Vision Technologies) for bright-field images. Snapshots (20×; tiff files) were taken from the central striatum. Colour images were converted to black/white images. Optical density of striatal DARPP32 immunohistochemistry (DAB staining) was quantified by digital image analysis (CellF; Soft Imaging Systems/Olympus) in each 3 sagittal section of two 8 months old wildtype, six 8-month-old HdhQ150 HET and six 8-month-old HdhQ150 HOM mice. In addition, each three frozen sagittal sections of four 10-month-old wild type and five 10-month-old HdhQ150 HET mice were analysed for striatal DARPP32 immunoreactivity by digital image analysis (CellF; Soft Imaging Systems/Olympus) of sagittal brain sections stained with a fluorescent (Alexa 594) secondary antibody. Mean values of staining intensities were statistically evaluated by Mann-Whitney U-test to analyse significance of differences.

### Digital image analysis and quantification of mHtt deposit-load

Snapshots (20×, tiff files) of MW8 immunofluorescence stainings counterstained with DAPI were taken from the central striatum and from the hilar region of the dentate gyrus. The %-age area covered by MW8 immunoreactive deposits was quantified in the respective areas by digital image analysis (CellF; Soft Imaging Systems/Olympus) of 3 sagittal sections per animal and six 8-month-old mice for each genotype (HdhQ150 HET and HdhQ150 HOM). In the striatum the %-age area covered by MW8 immunoreactive deposits (mainly NIIs) was normalized to the %-age area of DAPI stained nuclei. Mean values of staining intensities were statistically evaluated for significance of differences using the Mann-Whitney U-test.

### Retina histology

Eyes were fixed overnight in Davidson's solution (20% of a 37% formalin solution; 35% ethanol absolute; 10% glacial acid) and rinsed in 50% ethanol. The lenses were carefully removed and the fixed eye cups embedded in paraffin according to standard procedures. For general histology 3 µm retina paraffin sections (cross sectional plane) were de-waxed and stained with haematoxylin using an automated procedure (tissue stainer TST44C; Medite). To investigate Mueller glia cell activation, paraffin sections of Davidson's fixed retinas were subjected to automated GFAP immunohistochemistry (Discovery^XT^ technology).

### Western blotting

Striatum was isolated, snap frozen and stored at −80°C. Protein homogenates were prepared by homogenizing the striatum in 10×v/w of lysis buffer (PBS, 1% Triton X-100, complete protease inhibitor (Roche) and Pierce phosphatase inhibitor), using Precellys equipment (rotation speed: 5500 rpm, number of cycles: 2×30 sec, pause: 10 sec). Total homogenates were frozen at −80°C. For SDS PAGE gels, total homogenates were diluted 1∶10 or 1∶20 and 10 and 4 µl of extracts were loaded on 4–12% NUPAGE Bis-Tris gradient midi gels (Invitrogen) with MES SDS running buffer (Invitrogen) for 1 h at 180 V or 3–8% Tris-Acetate midi gels (Invitrogen) with Tris-acetate running buffer (Invitrogen) for 1.5 h at 130 V. For Western blotting, either a semi dry transfer system (BIO-RAD) was used with 2×Transfer buffer (Invitrogen) + 20% MeOH or TransBlot Turbo (Bio-Rad) according to the protocol of the manufacture. For semi-wet blotting transfer was for 1,5 h at constant voltage (20 V). Protein was transferred to PVDF membranes and blocked in 1∶1 dilution of PBS and Odyssey blocking buffer for 1 h. After blocking primary antibodies were applied for 16 h and membranes were incubated at 4°C. Antibody dilutions used: rabbit monoclonal DARPP-32 19A3 (Cell Signaling) 1∶1000–1∶4255; mouse anti-huntingtin 2B7, 4.8 mg/ml, 1∶1500; mouse anti-huntingtin MW1, 2.3 mg/ml, 1∶1500; loading control: mouse anti-ß-actin (Sigma) 1∶50000 and mouse anti-tubulin (Sigma) 1∶50000). After washing the membranes in a 1∶1 dilution of PBS and Odyssey blocking buffer, the membranes were incubated with secondary antibodies that included IRDye 800CW anti-rabbit IgG and either Alexa Fluor 680 F(ab’) fragments of goat anti-mouse or IRDye 680LT goat anti-mouse (dilution for all was 1∶5000). After 1 h at room temperature, bound antibodies were visualized using Odyssey infrared-imaging system (LI-COR) according to instructions provided by the manufacture. Western blot data were quantified using LI-COR software. Protein molecular weight marker was used from Odyssey. The monoclonal antibody MW1 was developed by Paul Patterson [34], [35] and obtained from the Developmental Studies Hybridoma Bank developed under the auspices of the NICHD and maintained by The University of Iowa, Department of Biological Sciences, Iowa City, IA 52242. The monoclonal anti-huntingtin 2B7 antibody was developed by Novartis and described previously [36].

### Statistical Analysis

Digital image analyses were statistically evaluated using the Mann-Whitney U-test and Western blot data using the one-way ANOVA Holm-Sidak's multiple comparisons test.

## Results

### Doubling *mHtt* gene-dose dramatically accelerates mHtt deposition

**Figure 1:**
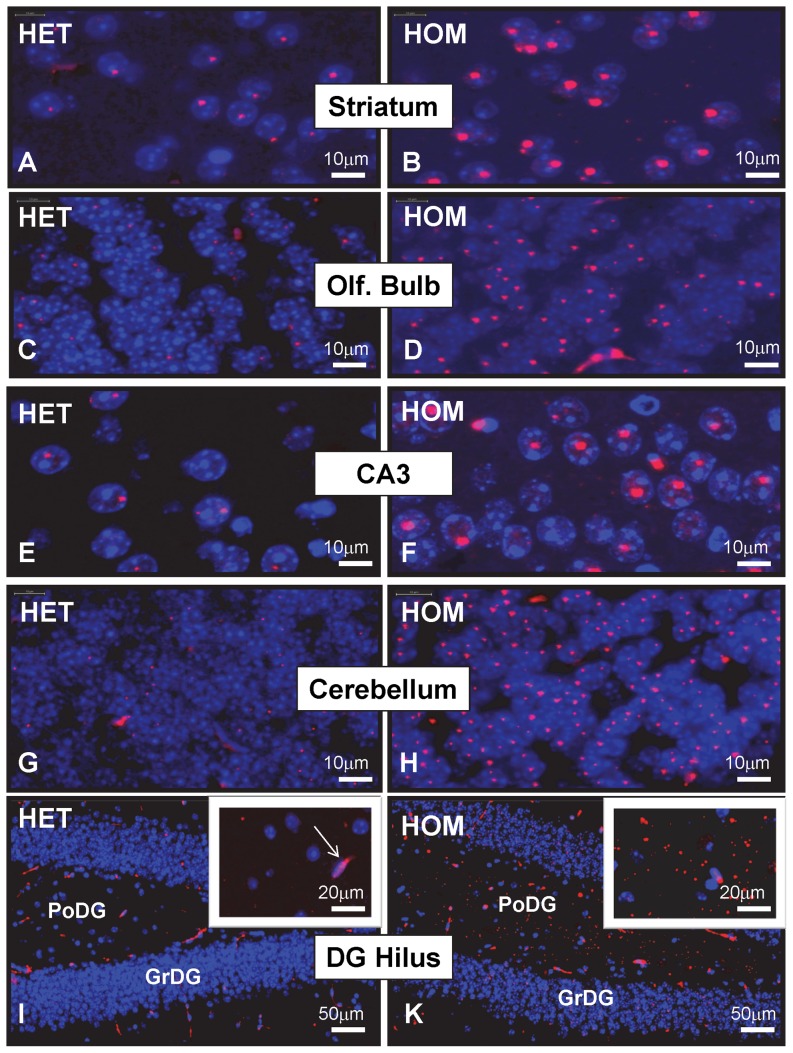
MW8^+^ mHtt aggregates in brain regions of HdhQ150 mice. MW8 immunofluorescence (in red) in frozen sections of 8-month-old HdhQ150 HET (A, C, E, G, I) and HdhQ150 HOM mice (B, D, F, H, K). Numerous neuronal intra-nuclear inclusions (NIIs) are visible in striatum (A, B), olfactory bulb (C, D), the CA3 region of the hippocampus (E, F) and in cerebellum (G, H). NIIs are much larger in HOM (B, D, F, H) as compared to HET mice (A, C, E, G). The dentate gyrus of HdhQ150 HOM mice (K) shows numerous extra-nuclear mHtt aggregates in the polymorph layer (PoDG) and large inclusions in the granular layer (GrDG). Such deposits are rare in the dentate gyrus of age-matched HdhQ150 HET mice (I). The large, elongated and irregularly shaped structures are blood vessels (e.g. see arrow in insert of I). These are non-specifically stained due to cross-reactivity of the secondary antibody with mouse IgG. The images are representative of results obtained from 6 HdhQ150 HOM and 6 HdhQ150 HET mice. Sections were counter-stained with DAPI (blue).

Western blot analyses were conducted to confirm that HdhQ150 HOM mice lack wildtype mHtt. Also, mHtt levels were increased in HOMs as compared to HETs (**Fig. S1**) as shown previously using FRET and SEC-FRET methods [17]. To compare NII and extra-nuclear aggregate-load in HdhQ150 HOM and HET mouse brains, mHtt deposits were detected with MW8, an antibody that recognizes a distinct epitope in mHtt aggregates [35]. Previous immunohistochemical analyses have shown that the number and size of MW8^+^ NIIs in the HdhQ150 HET mouse brain gradually increased with age and most prominently in the striatum [17]. In HdhQ150 HET mice this process started by 4 months of age, and it was strongly exacerbated in HdhQ150 HOM mice [17]. Here we show that by 8 months of age, NIIs are detectable in many additional brain regions such as the olfactory bulb, the hippocampus and the cerebellum. In all these regions, aggregate load was much greater in HdhQ150 HOM as compared to HET mice (**Fig. 1**). In some regions such as the brainstem, the majority of neurons lacked NIIs but exhibited varying numbers of much weaker stained small extra-nuclear aggregates. Also these types of aggregates were more abundant in HdhQ150 HOM as compared to HET mice (**Fig. S2**). Note that MW8 immunoreactivity in HdhQ150 mice is predominantly intra-nuclear and contrasts with findings in R6/2 mice, where MW8 stains both cytoplasmic and NIIs in all affected brain regions (**Fig. S3**). In HdhQ150 mice, one region did show an abundant load of extra-nuclear aggregates - the polymorph layer (hilus) of the dentate gyrus (PoDG). In this region extra-nuclear aggregates were very abundant in 8-month-old HdhQ150 HOM mice whereas their load in HET mice was still very low at this age (**Fig. 1** **I, K**). The PoDG also showed the highest density of extra-nuclear aggregates in R6/2 mice (**Fig. S4**). The aggregates in PoDG deserve special attention when assessing therapeutic strategies because extra-nuclear aggregates seem the more common type in the brain of HD patients [7]. In conclusion, doubling *mHtt* gene-dose dramatically exacerbated mHtt aggregate deposition in HdhQ150 HOM mice and this feature should greatly facilitate the preclinical assessment of potential therapeutics and genetic modifiers for their capability to lower extra-nuclear (PoDG) and intra-nuclear aggregate load.

### *mHtt* gene-dose impacts on DARPP32 levels

**Figure 2:**
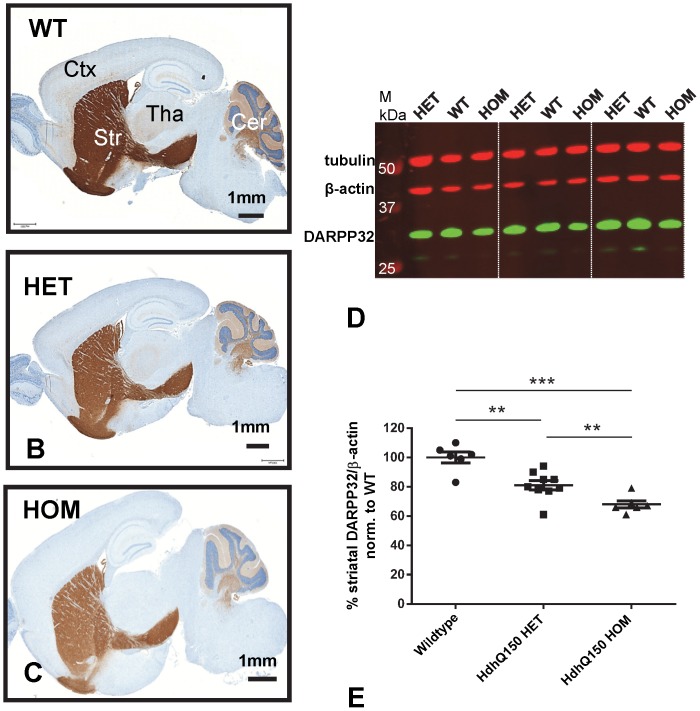
mHtt gene-dose impacts on DARPP32 protein levels. DARPP32 immunostaining in sagittal mouse brain sections of 8-month-old wildtype (A), HdhQ150 HET (B), and HdhQ150 HOM mice (C). Staining was performed by automated paraffin immunohistochemistry using the Ventana Discovery XT technology and DAB as chromogen. Overall DARPP32 staining intensities clearly decline from wildtype (A) to HdhQ150 HET (B) and HOM mice (C), and in all DARPP32^+^ brain regions including striatum (Str), cortex (Ctx), thalamus (Tha), and cerebellum (Cer). The images are representative of results obtained from 2 WT, 6 HdhQ150 HET and 6 HdhQ150 HOM mice. (D) Representative western blot of DARPP32 protein levels in 6-month-old wildtype (WT), HdhQ150 HET and HOM mice, 3 of each. (E) Quantification of Western blot signals (normalized to wildtype and loading control β-actin) revealed a highly significant reduction of DARPP32 levels in HdhQ150 striatum, as compared to wildtype striatum (n = 6, p<0.005). The difference between HdhQ150 HET (n = 9) and HdhQ150 HOM (n = 6) DARPP32 levels was also statistically significant (p<0.01). Quantification results are the average of three independent Western blots. A second control (tubulin) shows that DARPP32 changes are not due to differences in loading. Statistics: One-way ANOVA, Holm-Sidak's multiple comparisons test

Dopamine- and cyclic AMP-regulated phosphoprotein, 32 kDa (DARPP32) is a cytosolic protein that is highly enriched in striatal medium spiny neurons (MSNs). The protein is a pivotal integrator of dopamine signaling in MSNs neurons [37]. DARPP32 is one of several genes downregulated in the HD striatal transcriptome, also in HD animal models, and these effects on transcription seem an essential feature of HD pathogenesis [38], [39], [40]. Here we tested, whether there is a correlation between DARPP32 levels and *mHtt* gene-dose. At 8 months of age, the vast majority of DARPP32 MSNs neurons in HdhQ150 mice contained large NIIs (**Fig.1A, B**). DARPP32 levels were reduced in a *mHtt* gene-dose dependent fashion as shown by immunostainings of whole brain sagittal sections (**Fig. 2A-C**) and Western blot analysis (**Fig. 2** **D, E**). There was a greater reduction in the optical density of DARPP32 immunostaining in MSNs of HdhQ150 mice carrying two *mHtt* alleles as compared to mice carrying one *mHtt* allele. Quantitative image analysis of paraffin sections stained using automated immunohistochemistry revealed, as compared to wildtype mice, an overall decrease in DARPP32 staining intensities of ∼10% in 8-month-old HdhQ150 HET mice and ∼30% in 8-month-old HdhQ150 HOM mice (**Fig. S5** **A-D**). The difference in DARPP32 staining intensity between HOM and HET mice was statistically highly significant (Mann-Whitney U-test, p < 0.01). Using a different manual immunofluorescence staining procedure in frozen sections, we confirmed reduced DARPP32 levels in HdhQ150 mice when comparing striatum of wildtype and 10-month-old HdhQ150 HET mice (**Fig. S5 E -G**). Western blot analysis of 6-month-old wild type, HdhQ150 HET and HdhQ150 HOM mice revealed that the overall levels of DARPP32 in the tissue were reduced by ∼19% in HdhQ150 HET and by ∼32% in HOM mice (**Fig. 2** **D, E**). These differences between wildtype and HdhQ150 HET and between HdhQ150 HET and HOM mice were statistically highly significant.

**Figure 3:**
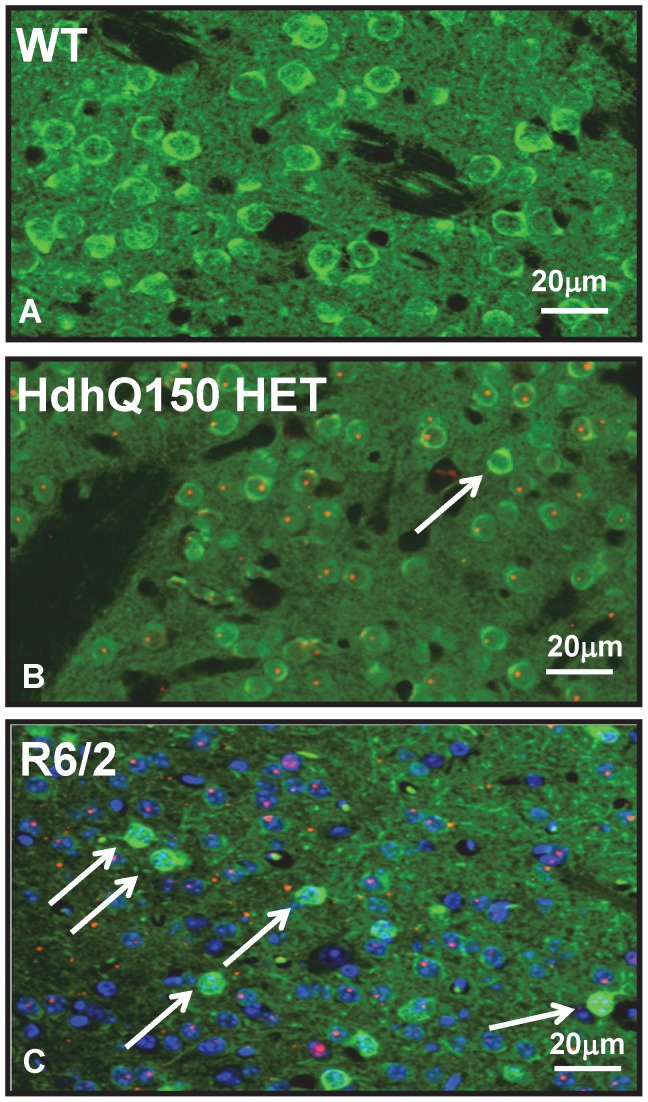
mHtt aggregates in DARPP32^+^ striatal neurons of HdhQ150 and R6/2 mice. Images represent paraffin sections of striatum, double stained (immunofluorescence) with anti-DARPP32 (green) and MW8 to visualize neuronal intra-nuclear inclusions (NIIs; shown in red). Shown are central regions of the striatum of a 10-month-old wildtype mouse (WT; A), a 10-month-old HdhQ150 HET mouse (B) and a 12-week-old R6/2 mouse (C). NIIs are visible in most MSNs but absent in neurons with high DARPP32 staining signals (arrows). The images are representative of results obtained from 3 WT, 3 HdhQ150 HET and 3 R6/2 mice.

The *mHtt* gene-dose dependent decrease in DARPP32 staining in HdhQ150 mice was not restricted to MSNs in the striatum. It was also seen in DARPP32^+^ neurons located in thalamus, cerebellum and cortex (**Fig. 2A-C**) and similar observations were made in 10-week-old R6/2 mice (**Fig. S6**). We noted, that the HdhQ150 striatum contained also a low number of MSNs with high DARPP32 staining intensities (DARPP32^high^) and devoid of NIIs (**Fig. 3B**). Whether these cells represent extreme case examples of an inverse relationship between mHtt dose and DARPP32 is not known. DARPP32^high^ MSNs devoid of aggregates were even more numerous in the striatum of R6/2 mice (**Fig. 3C**). Taken together, these data demonstrate that there is a clear inverse relationship between *mHtt* gene-dose and DARPP32 levels measured by Western blot or immunohistochemistry.

### The HdhQ150 mouse retina contains mHtt deposits

**Figure 4:**
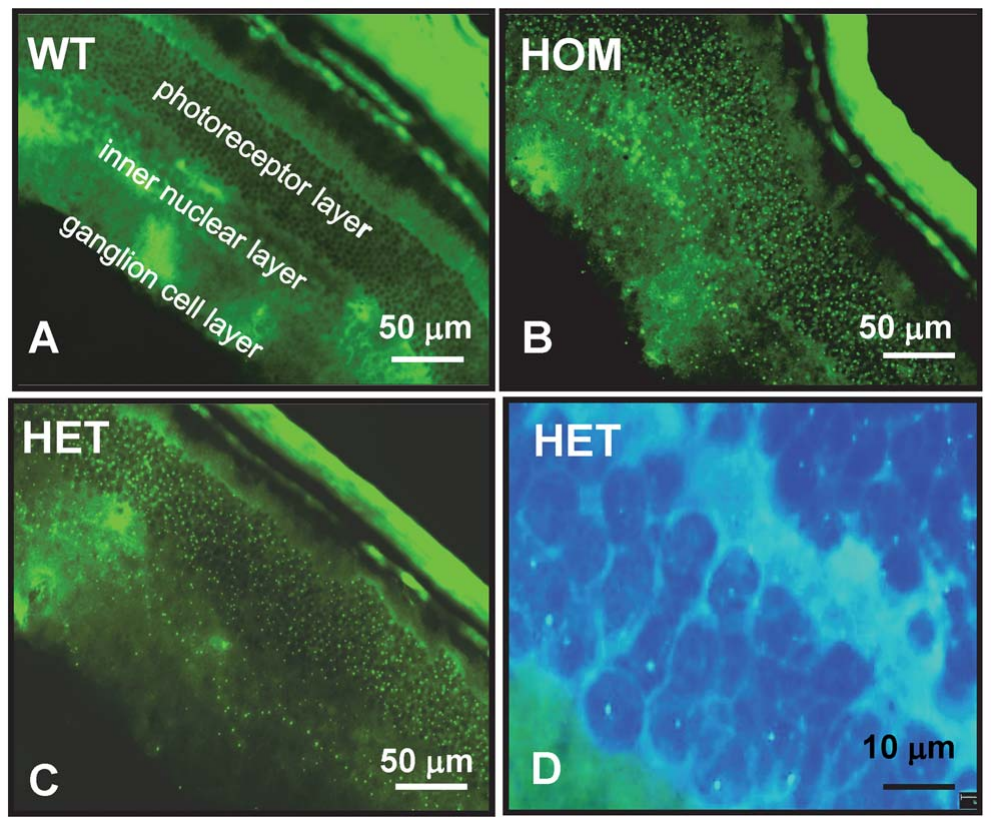
mHtt aggregates in the HdhQ150 mouse retina. Images show MW8 immunofluorescence in frozen sections of the retina of a wildtype mouse (A), a 6-month-old HdhQ150 HOM mouse (B), and a 10-month-old HdhQ150 HET mouse (C). Aggregates (green dots) are visible only in the HdhQ150 mouse retinas where these are present in all neuronal cell layers and cell types including photoreceptors, inner nuclear and ganglion cells. No aggregates were detected in non-neuronal cell layers such as choroid or pigment epithelium. Aggregate size and number is similar in 6-month-old HdhQ150 HOM and 10-month-old HdhQ150 HET mouse retinas. (D) High magnification image of C showing that retinal neuron mHtt deposits are mainly intra-nuclear (NIIs). MW8 staining results at higher magnification show mHtt inclusions (green dots) on a blue background of DAPI-stained nuclei. Large irregularly shaped green areas are due to non-specific cross-reactivity of the secondary antibody with mouse IgG. Images are representative of results obtained from 4 HdhQ150 HET and 3 HdhQ150 HOM mice.

Previous work in some transgenic HD models has shown that changes occur in the retina [41], [42], [43] but the clinical significance of these findings remain to be elucidated. Here, we analysed and compared aggregate load in retinas of 6-month-old HdhQ150 HOM and 10-month-old HdhQ150 HET mice. Interestingly, NIIs were found in all neuronal cell layers and neuronal cell types of the retina including ganglion cells, inner nuclear cells and photoreceptors (**Fig. 4**). Aggregates were not found in non-neuronal cell types such as pigment epithelial and choroid cells. NIIs in retinal neurons of 6-month-old HdhQ150 HOM mice had already reached the size of NIIs seen in the retina of 10-month-old HdhQ150 HET mice (**Fig. 4**). The large number of deposits seen in the retina of 6-month-old HdhQ150 HOM mice suggests that the deposition of mHtt inclusions is greatly exacerbated in retinas of HOM as compared to HET mice. We did not detect gross morphological changes in HdhQ150 retinas in contrast to 12-week-old R6/2 mouse retinas (**Fig. S7**), which had a waved and thinned photoreceptor layer as reported by others [41]. Finally, GFAP immunostainings revealed no signs of Müller glial cell activation, neither in HdhQ150 nor in R6/2 mice (**Fig. S7**). In conclusion, our results demonstrate that retinal neurons in HdhQ150 mice develop an abundant aggregate-load which appears to be influenced by *mHtt* gene-dose like in brain neurons. Furthermore, our findings show that retinal NIIs are not specific to HD model mice expressing human exon-1 transgenes (R6) lines.

### Increasing *mHtt* gene-dose worsens neuroinflammation

**Figure 5:**
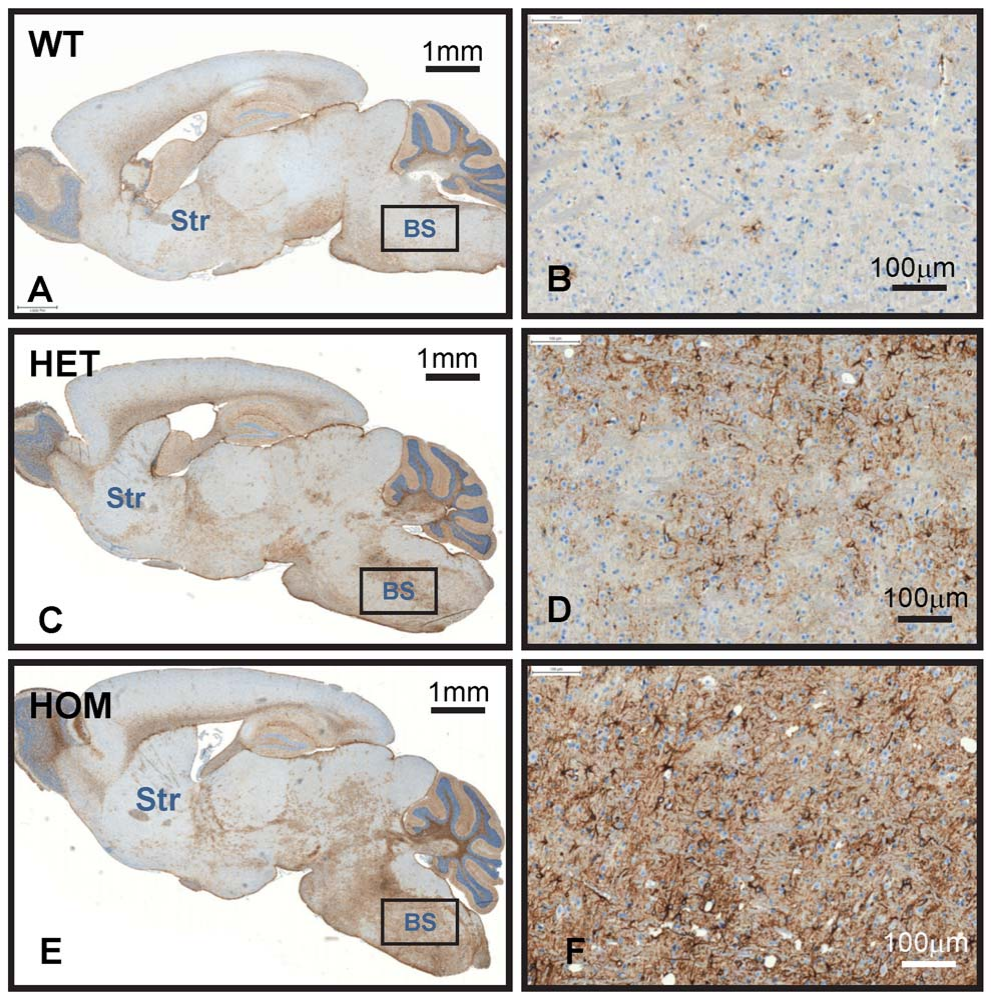
GFAP^+^ astroglial cells in the hind brain of HdhQ150 mice. Images show the results of GFAP immunohistochemistry on sagittal brain sections of 8-month-old wildtype (A, B), HdhQ150 HET (C, D), and HdhQ150 HOM mice (E, F). Staining was performed by automated immunohistochemistry using the Ventana Discovery XT technology and DAB as chromogen. As compared to wildtype (A, B), GFAP staining is dramatically increased in the brainstem (BS) of HdhQ150 HET mice (C, D) and the increase is much more pronounced in HdhQ150 HOM mice (E, F). Note that the striatum (Str) in HdhQ150 mice is largely devoid of activated astroglia. The images are representative of results obtained from 2 wildtype, 6 HdhQ150 HOM and 6 HdhQ150 HET mice.

**Figure 6:**
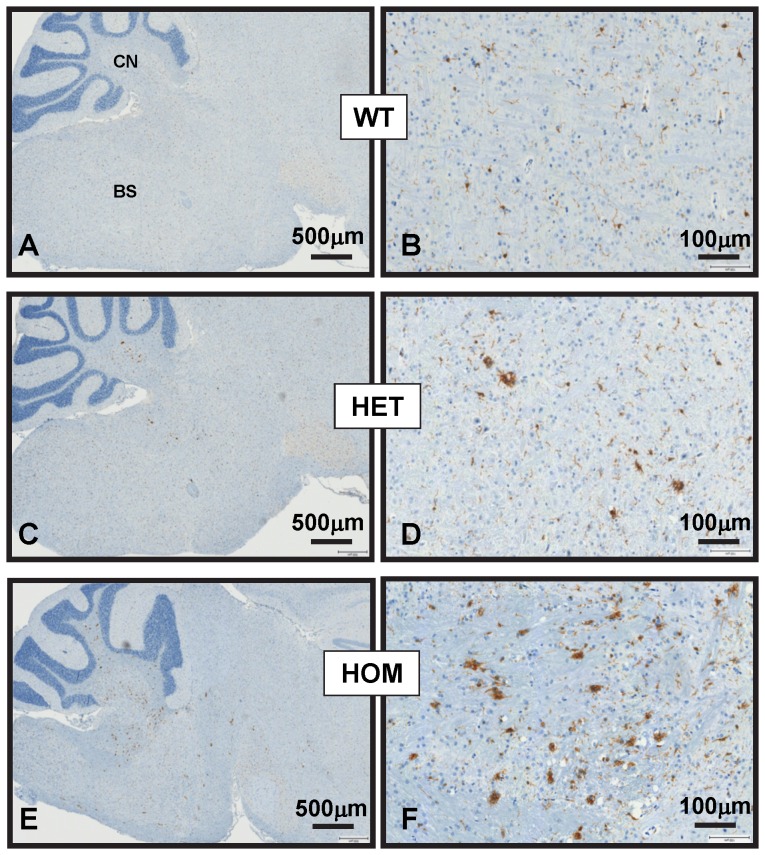
Iba 1^+^ microglial cells in the hind brain of HdhQ150 mice. Images represent Iba1 immunohistochemical staining results in sagittal brain sections of 8-month-old wildtype (A, B), HdhQ150 HET (C, D), and HdhQ150 HOM mice (E, F). Staining was performed by automated immunohistochemistry using the Ventana Discovery XT technology and DAB as chromogen. As compared to wildtype (A, B), Iba 1 staining is more pronounced in brainstem and cerebellar nuclei of HdhQ150 mice and markedly increased in HdhQ150 HOM as compared to HdhQ150 HET mice. The brainstem and cerebellar nuclei of HdhQ150 mice contain numerous large Iba1^+^ cells with an ameboid morphology reminiscent of activated microglia. The images are representative of results obtained from 2 wild type, 6 HdhQ150 HOM and 6 HdhQ150 HET mice.

Histopathological manifestations of neuroinflammation in brain have been documented in many neurodegenerative diseases with proteinopathy including presymptomatic and symptomatic HD [44]. Whether mutant huntingtin promotes neuroinflammation directly or indirectly and whether it drives the process or occurs in response to neuronal damage or death remains to be elucidated. Here, we investigated whether HdhQ150 mouse brains exhibit signs of neuroinflammation in relation to *mHtt* gene-dose. Immunostainings were performed with antibodies that selectively mark astrocytes (anti-GFAP) and microglial cells (anti-Iba1). Iba1 (ionized calcium-binding adaptor molecule 1) is a 17-kDa calcium binding EF hand protein, specifically expressed in macrophages and microglial cells and widely used to identify activated microglia. Glial-fibrillary acidic protein (GFAP) is a cytoplasmic filamentous protein and a component of the cytoskeleton in astrocytes. As compared to wildtype mice we found, that brainstem and cerebellar nuclei in 8-month-old HdhQ150 HET and HOM mice showed a significant increase in the number of GFAP^+^ astrocytes (**Fig. 5**) and Iba1^+^ microglia (**Fig. 6**). Many Iba1^+^ microglial cells displayed an amoeboid morphology characteristic of activated microglia. The increase in glial inflammatory markers was much more pronounced in 8-month-old HdhQ150 HOM as compared to HET mice (**Fig. 5** **and** **6**). Surprisingly, activated glial cells were not evident in brain regions with high aggregate load such as the striatum. In contrast, in the two brain areas (brainstem and cerebellar nuclei) with pronounced glial cell activation, most neurons lacked NIIs. No glial cell activation or mutant mHtt deposition was observed in the brainstem of 4-month-old HdhQ150 HOM and HET mice (data not shown). Altogether, these findings suggest that *mHtt* gene-dose but not local NII burden correlates with the magnitude of glial cell activation in the HdhQ150 brain.

## Discussion

Here we demonstrated that doubling *mHtt* gene-dose in HdhQ150 mice has a massive impact on the deposition of NIIs and extra-nuclear mHtt aggregates. It also markedly reduced DARPP32 levels below those detected in mice carrying a single *mHtt* allele and it exacerbated the expression of glial neuroinflammatory markers. All these changes were observed at an age, well before behavioural symptoms have been reported to emerge in HdhQ150 mice [18]. Together with recently developed methods to quantify soluble and insoluble mHtt [16], [17], [45], the here described robust *mHtt* gene-dose related changes in neuropathological markers should facilitate future assessments of novel therapeutic strategies in the HdhQ150 mouse HD model.

We confirmed earlier observations [14], [22], showing a marked and progressive accumulation of NIIs in the HdhQ150 mouse brain. This phenomenon was most pronounced in the striatum, a brain region that is severely and early affected in HD patients [46], [47] although aggregate deposition in the striatum seems less pronounced than in the HD cortex [7]. We previously showed that the MW8 antibody detected numerous large NIIs in striatal MSNs of 4-month-old HdhQ150 HOM mice [17], and which seems in line with results described by Baldo et al. [16] and Bayram-Weston et al. [22]. Others reported aggregates appearing not earlier than at 8 months [14]. These discrepancies may relate in part to different methodologies and antibodies used by us and other investigators to detect mHtt aggregates. It seems less likely, that the different results are due to major variations in CAG repeat-length of the *mHtt* allele. However, they could relate to the different mouse strain genetic backgrounds used by Woodman et al. [14] (C57BL/6×129Ola) and us in this study (C57BL/6). Genetic background can modify mHtt accumulation as shown by Lloret et al. [48] who reported, that NII formation in HdhQ111 knock-in mice was faster in C57BL/6 as compared to 129Sv mice.

The vast majority of mHtt aggregates in the HdhQ150 mouse brain were NIIs with the exception of one brain region, the PoDG, that also in R6/2 mice displayed an extraordinary high density of extra-nuclear aggregates. However, the translational value of these findings remains unknown and deserves great caution because of fundamental differences between the rodent and human DG [49]. In HdhQ150 and also R6/2 mice, the extra-nuclear aggregates in the PoDG may reside in a number of different cell types. The PoDG harbours granule-cell axonal plexuses, mossy cells, different GABAergic interneurons, extrinsic afferent connections of septal cholinergic and GABAergic neurons and noradrenergic inputs from locus coeruleus [49]. Elucidating this complex picture would go far beyond the scope of this study. Nonetheless, quantifying aggregate load in the PoDG may greatly facilitate the characterization of mechanisms and drug candidates that can specifically lower extra-nuclear aggregate-load, both for mHtt in HdhQ150 and for mHTT in R6/2 mice. This may be important since extra-nuclear aggregates in HD patients seem to be much more common [7]. Also, the PoDG is a well circumscribed structure with clearly defined borders which facilitates quantification of aggregate-load in brain sections using digital image analysis.

To our knowledge, the present study in HdhQ150 mice shows for the first time that changes in DARPP32 are exquisitely sensitive to *mHtt* gene-dose. DARPP32 is critically involved in the regulation of MSN physiology [50]. Using Western blot analysis, we showed that one *mHtt* allele reduced DARPP32 protein levels in 6-month-old HdhQ150 mice by ∼19% as compared to ∼32% when two *mHtt* alleles were present. This dysregulation of DARPP32 in the striatum appears to occur before motor deficits emerged by 70 weeks [18] and before others reported reduced DARPP32 mRNA levels [14]. Also in CAG140 knock-in mice, reduced DARPP32 immunostaining signals have been reported to occur late by 12 months [21]. Our findings in HdhQ150 mice seem in line with observations in R6/2 mice where DARPP32 is reduced by 4 weeks, well before the onset of behavioural phenotypes [51]. For comparison, R6/1 mice which develop disease much slower as compared to R6/2 mice, showed a downregulation of DARPP32 by 5 months [52]. The *mHtt* gene-dose dependent reduction of DARPP32 staining intensity in HdhQ150 mice was also observed in neurons located in thalamus, cerebellum and cortex. This further emphasizes the inverse relationship seen between *mHtt* gene-dose and DARPP32 levels in neurons, irrespective of their anatomical location in the brain. Also, these findings do not concur with the hypothesis that DARPP32 downregulation is specific to striatal MSNs [51]. We also noted that a small subset of DARPP32^high^ MSNs lacked mHtt NIIs in HdhQ150 and mHTT NIIs in R6/2 mice. It is unclear whether these cells express less mutant huntingtin. Alternatively, their physiological integrity is perhaps less compromised and these cells may still be able to clear aggregates. DARPP32 is regulated by BDNF/TrkB signaling [53] and a reduced supply of BDNF via corticostriatal connections is thought to play a role in the pathogenesis of HD [54]. Therefore, the changes in DARPP32 levels likely reflect also early changes in corticostriatal circuit integrity and perhaps, at the level of single MSNs, a local afferent supply of BDNF. Irrespective, DARPP32 in C57BL/6 HdhQ150 mice seems a sensitive and early indicator of mHtt dose-related changes in MSN integrity.

Our findings that retinal neurons in HdhQ150 mice develop aggregates raises an important question, namely whether these findings are translational to the HD retina. Histopathophysiological changes have been reported in the retina of some transgenic HD models [41], [42], [43]. Here we showed for the first time, that HdhQ150 mice accumulate massive numbers of mHtt NIIs in retinal neurons. This finding demonstrates that retinal NIIs are not specific to HD mouse models transgenic for human *mHTT* exon-1. We confirmed that R6/2 mouse retinal neurons contained NIIs. Also, the R6/2 retina showed a waved and thinned photoreceptor layer as described by others [41]. However, similar histopathological changes were not observed in the HdhQ150 mouse retina. One study has reported a lack of mHTT deposits in the post mortem retina of a 69 year old HD patient with a 28-year history of clinically manifest HD [55]. This report described a single and perhaps unusual case and additional studies will be required to clarify whether NIIs and/or extra-nuclear aggregates occur in the HD retina. This might have clinical relevance as the eye could offer unique opportunities for non-invasive monitoring of mHTT deposits and associated retinal changes.

Whether glial cell activation in patients and HD models is beneficial, harmful or disease-stage dependent remains poorly understood. Studies in patients suggest that in particular microglial cell activation may play a role in the pathophysiology of HD. PET studies have shown a correlation between the level of striatal microglia activation, disease severity and loss of raclopride binding in the striatum [56], [57], also in presymptomatic HD brains. These studies suggested widespread increases in the activation of microglia early in the disease, also in sites distant from regions primarily affected [58], [59]. Curiously, in HdhQ150 mice we did not detect glial cell activation in striatum where aggregate-load was high. In contrast, areas including brainstem and cerebellar nucleus showed a pronounced increase in glial activation markers whereas neurons in these regions were largely devoid of large aggregates. HdhQ150 mice showed no obvious signs of neuronal atrophy or axonal dystrophy as analysed by neurofilament staining (not shown). Therefore, the observed local increase in glial inflammatory markers seems unrelated to aggregate-burden in neuronal cell bodies present in these regions. Perhaps it is related to mHtt-induced changes in remote but connected brain areas or special properties of local neurons, which might be able to dispose of mHtt and trigger a local glial cell response. Finally, it has been described that R6/2 mouse brains contain increased numbers of often dystrophic, ferritin-loaded microglial cells with high iron content, similar to microglial cell phenotypes seen in HD brains [60]. The R6/2 mice we analyzed also contained many ferritin-loaded microglial cells with mHtt deposits (not shown). However, we failed to detect such cells in HdhQ150 mice which could suggest, that perturbations in microglial iron metabolism may occur mainly during advanced stages of the disease. Our most important finding was, however, that the dramatic regional increase in glial inflammatory markers correlated with *mHtt* gene-dose although it did not correlate with local NII-burden. These and the other robust and striking mHtt dose-related changes described here should significantly facilitate future preclinical evaluations of mHtt-lowering therapeutic strategies in the HdhQ150 mouse HD model.

## Supporting Information

Figure S1 **Gene-dose related changes in mHtt expression and mHtt deposition.** Western blot analysis (A) illustrates the differences in the expression levels of full-length wildtype huntingtin (wtHtt) and full-length mutant huntingtin (mHtt) protein in the striatum of 2 wildtype, 2 HdhQ150 HET and 2 HdhQ150 HOM mice. The MW1 antibody is specific for mHtt while 2B7 detects both mHtt and wtHtt. (B) Digital image analysis of frozen sections stained with MW8 revealed a significant increase in mHtt NII load in striatal nuclei of HdhQ150 HOM as compared to HdhQ150 HET mice (p<0.01). Inclusion load is defined as the surface area (%) occupied by MW8^+^ inclusions and normalized to the surface area (%) of the DAPI-stained nuclei. (C) Similar analysis was conducted to show a highly significant increase (p<0.01) in the load of extra-nuclear aggregates in the PoDG. Statistical significance of the differences was confirmed using the Mann-Whitney U-test.
(TIF)

Figure S2 **MW8^+^ mHtt deposits in the HdhQ150 mouse brainstem.** Images show MW8^+^ mHtt deposits in paraffin sections of the brainstem of an 8-month- old HdhQ150 HET (A) and an 8-month-old HdhQ150 HOM mouse (B). For reference, a wildtype mouse brain section (WT) is shown that is completely devoid of MW8 staining. Most aggregates in brainstem are small and extra-nuclear. Their number is markedly higher in HdhQ150 HOM as compared to HET mice. The images are representative of results obtained from independent staining experiments of sections from 2 wildtype, 6 HdhQ150 HET and 6 HdhQ150 HOM mice.
(TIF)

Figure S3 **MW8^+^ mHtt deposits in cortex and striatum of R6/2 mice.** MW8^+^ neuronal intra-nuclear inclusions (NIIs), extra-nuclear aggregates and diffuse mHtt immunofluorescence (red) staining are seen in neurons located in cortex (A) and striatum (B) of a 10-week-old R6/2 mouse. NIIs and extra-nuclear aggregates are also shown in paraffin sections of a 10-week-old R6/2 mouse. These sections were processed using automated DAB immunohistochemistry. Counterstain is hematoxylin (C and D). Images are representative of independent staining experiments using 3 sections/animal and 3 animals/genotype.
(TIF)

Figure S4 **MW8^+^ mHtt aggregates in the dentate gyrus of R6/2 mice.** MW8^+^ aggregates are shown in the dentate gyrus of a 10-week-old R6/2 mouse to illustrate the high-load of extra-nuclear aggregates in the polymorph layer (PoDG) and the presence of NIIs in neurons of the granule cell layer (GrDG). The image is representative of independent staining experiments using 3 sections/animal and 3 animals/genotype.
(TIF)

Figure S5 **Reduced DARPP32 immunostaining in the HdhQ150 mouse striatum.** Images show a comparison of the different DARPP32 immunostaining intensities in the striatum of an 8-month-old wildtype (A), HdhQ150 HET (B) and HdhQ150 HOM mouse (C). (D) Quantitative evaluation of optical density (digital image analysis) revealed statistically significant reductions in DARPP32 staining intensities between HdhQ150 HET (n = 6) and HdhQ150 HOM (n = 6) mice (Mann-Whitney test: p<0.01). (E, F, G) Results are also shown for 10-month-old mice using a different method based on DARPP32 immunofluorescence. Staining intensities in striatum are shown comparing a 10-month-old wildtype (E) and a 10-month-old HdhQ150 HET mouse (F). (G) Digital image analysis of DARPP32 fluorescence intensities revealed significant reduction in DARPP32 signals of HdhQ150 HET (n = 5) as compared to wildtype mice (n = 4). Significance of differences was confirmed using the Mann-Whitney U-test (p<0.01).
(TIF)

Figure S6 **Reduced DARPP32 immunostaining signals in the R6/2 mouse brain.** Representative sagittal brain sections are shown of a 10-week-old wildtype (WT) and a 10-week-old R6/2 mouse stained for DARPP32. These show the dramatic reduction of DARPP32 staining signals in the R6/2 striatum (Str), the substantia nigra (SN) and the cerebellum (Cer) and the absence of DARPP32 signals in R6/2 thalamus (Tha) and cortex (Ctx). Images are representative of independent staining experiments using 3 sections/animal, 3 wildtypes and 3 R6/2 mice.
(TIF)

Figure S7 **Retina histology of HdhQ150 and R6/2 mice.** Images show GFAP staining in Davidson fixed eyes of an 8-month-old wildtype mouse (A), a 10-week-old R6/2 mouse (B), and a 10-month-old HdhQ150 HET mouse (C). None of the retinas displayed enhanced GFAP staining (indicator of Müller glia cell activation). The HdhQ150 HET retina (C) shows no obvious histological abnormalities whereas the photoreceptor layer in the R6/2 retina exhibits a waved structure. The images are representative of independent staining experiments using 3 sections/animal, 3 wildtypes, 4 HdhQ150 HETs and 3 R6/2 mice.
(TIF)
