# Supplementary figures and images for: Mutant Huntingtin Gene-Dose Impacts on Aggregate Deposition, DARPP32 Expression and Neuroinflammation in HdhQ150 Mice
Source: PLoS One. 2013 Sep 23;8(9):e75108. doi: 10.1371/journal.pone.0075108 (PMC3781050; doi:10.1371/journal.pone.0075108)

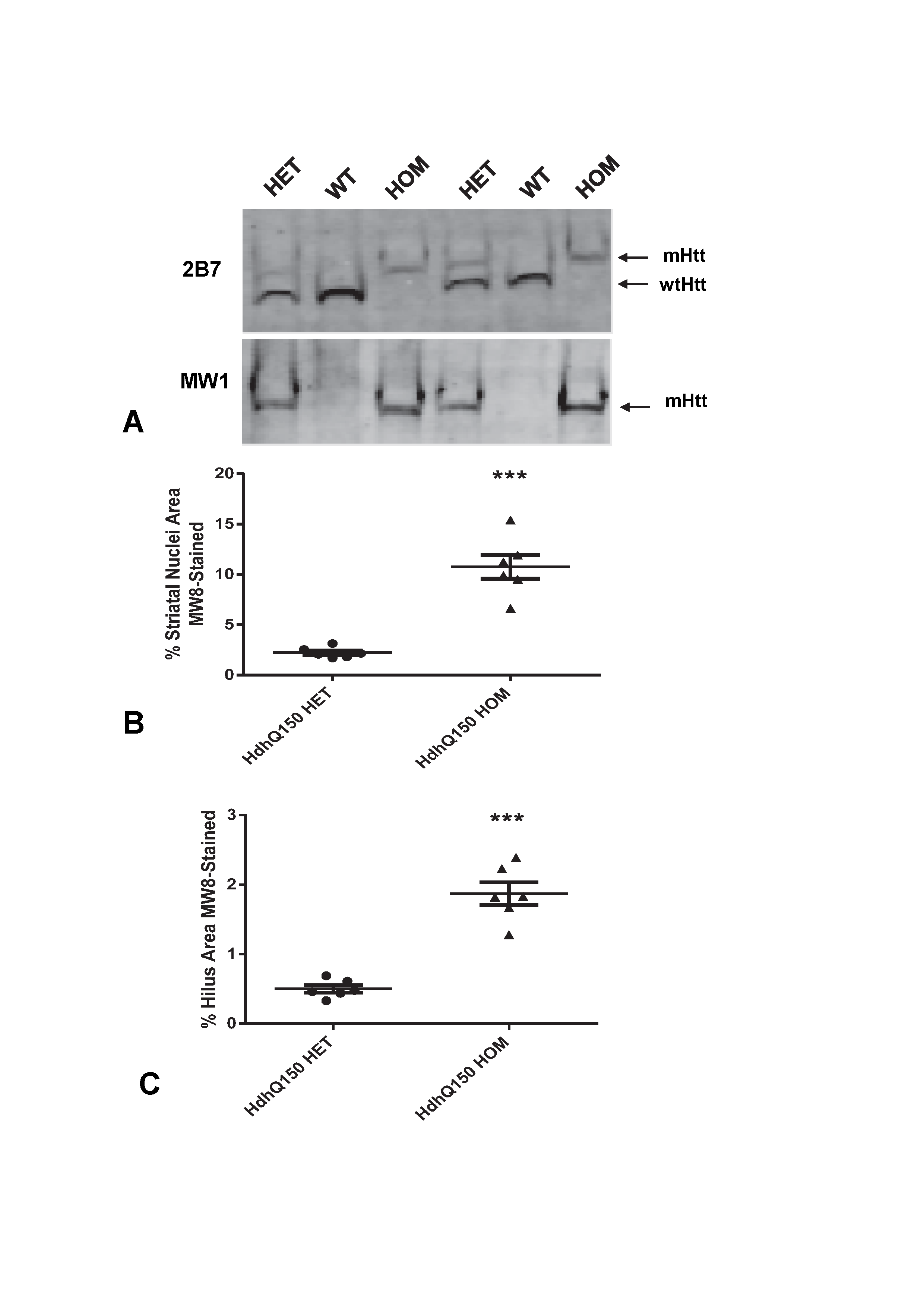

Supplement: Figure S1 — Gene-dose related changes in mHtt expression and mHtt deposition. Western blot analysis (A) illustrates the differences in the expression levels of full-length wildtype huntingtin (wtHtt) and full-length mutant huntingtin (mHtt) protein in the striatum of 2 wildtype, 2 HdhQ150 HET and 2 HdhQ150 HOM mice. The MW1 antibody is specific for mHtt while 2B7 detects both mHtt and wtHtt. (B) Digital image analysis of frozen sections stained with MW8 revealed a significant increase in mHtt NII load in striatal nuclei of HdhQ150 HOM as compared to HdhQ150 HET mice (p<0.01). Inclusion load is defined as the surface area (%) occupied by MW8+ inclusions and normalized to the surface area (%) of the DAPI-stained nuclei. (C) Similar analysis was conducted to show a highly significant increase (p<0.01) in the load of extra-nuclear aggregates in the PoDG. Statistical significance of the differences was confirmed using the Mann-Whitney U-test. (TIF) [file pone.0075108.s001.tif]

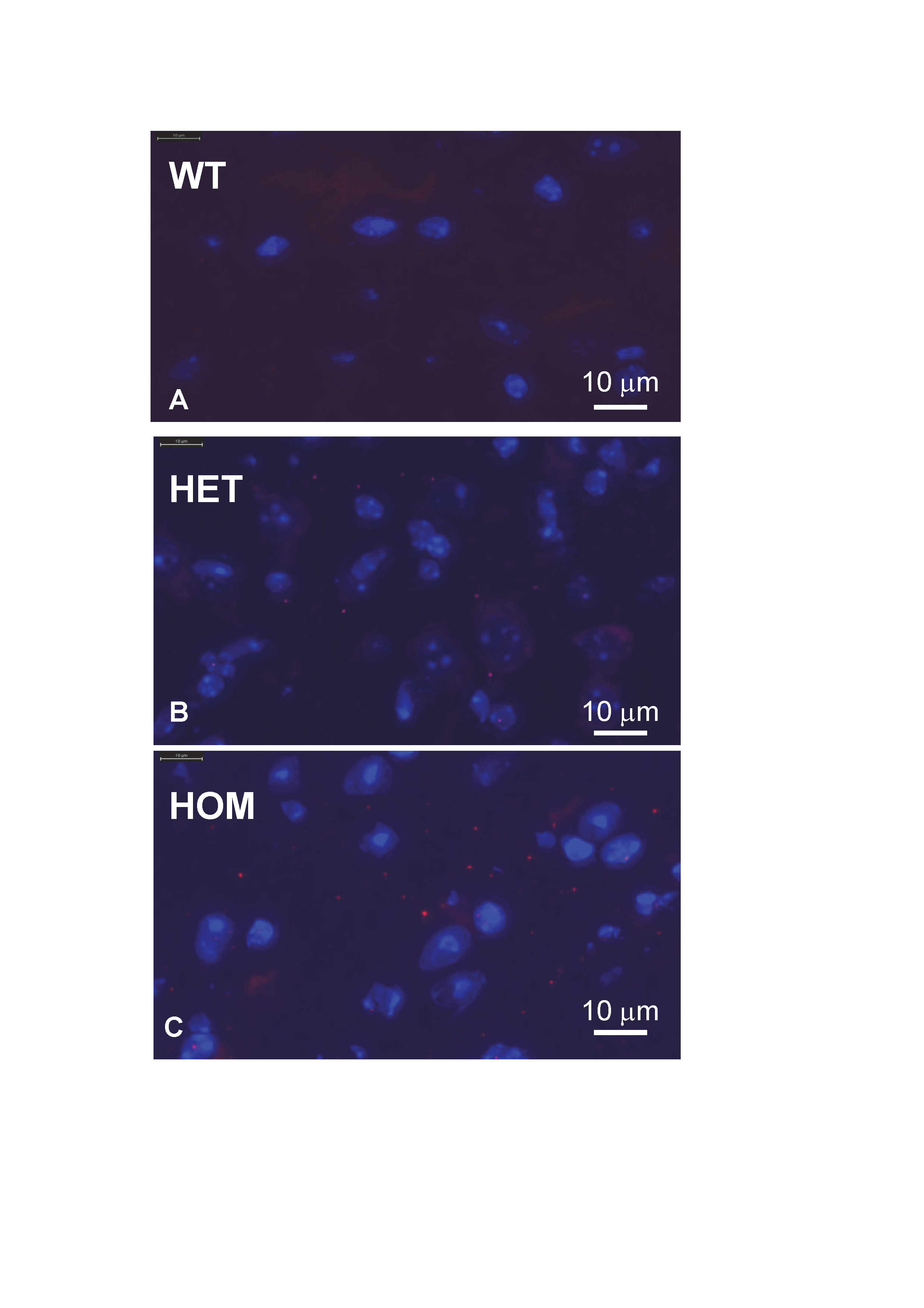

Supplement: Figure S2 — MW8+ mHtt deposits in the HdhQ150 mouse brainstem. Images show MW8+ mHtt deposits in paraffin sections of the brainstem of an 8-month- old HdhQ150 HET (A) and an 8-month-old HdhQ150 HOM mouse (B). For reference, a wildtype mouse brain section (WT) is shown that is completely devoid of MW8 staining. Most aggregates in brainstem are small and extra-nuclear. Their number is markedly higher in HdhQ150 HOM as compared to HET mice. The images are representative of results obtained from independent staining experiments of sections from 2 wildtype, 6 HdhQ150 HET and 6 HdhQ150 HOM mice. (TIF) [file pone.0075108.s002.tif]

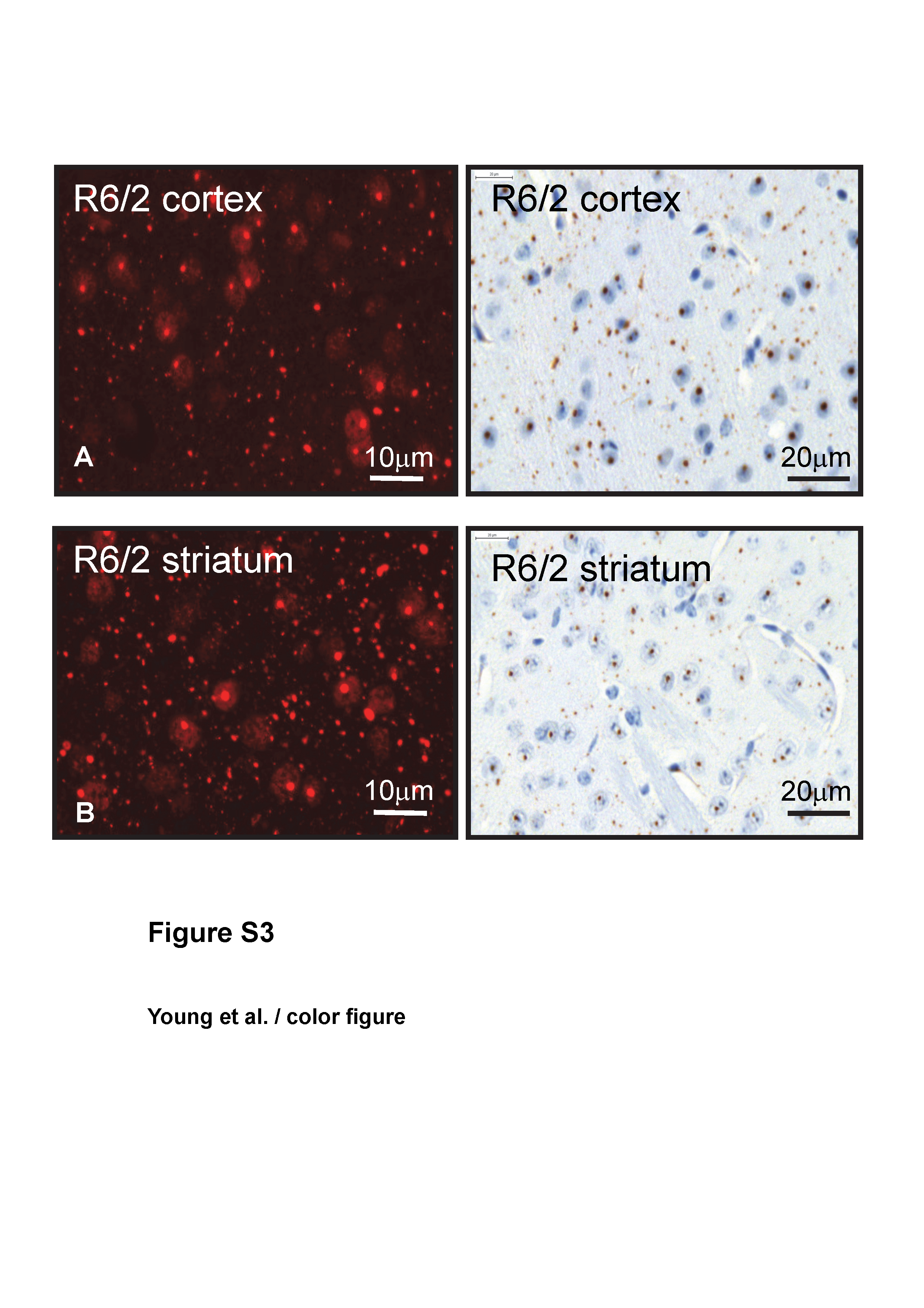

Supplement: Figure S3 — MW8+ mHtt deposits in cortex and striatum of R6/2 mice. MW8+ neuronal intra-nuclear inclusions (NIIs), extra-nuclear aggregates and diffuse mHtt immunofluorescence (red) staining are seen in neurons located in cortex (A) and striatum (B) of a 10-week-old R6/2 mouse. NIIs and extra-nuclear aggregates are also shown in paraffin sections of a 10-week-old R6/2 mouse. These sections were processed using automated DAB immunohistochemistry. Counterstain is hematoxylin (C and D). Images are representative of independent staining experiments using 3 sections/animal and 3 animals/genotype. (TIF) [file pone.0075108.s003.tif]

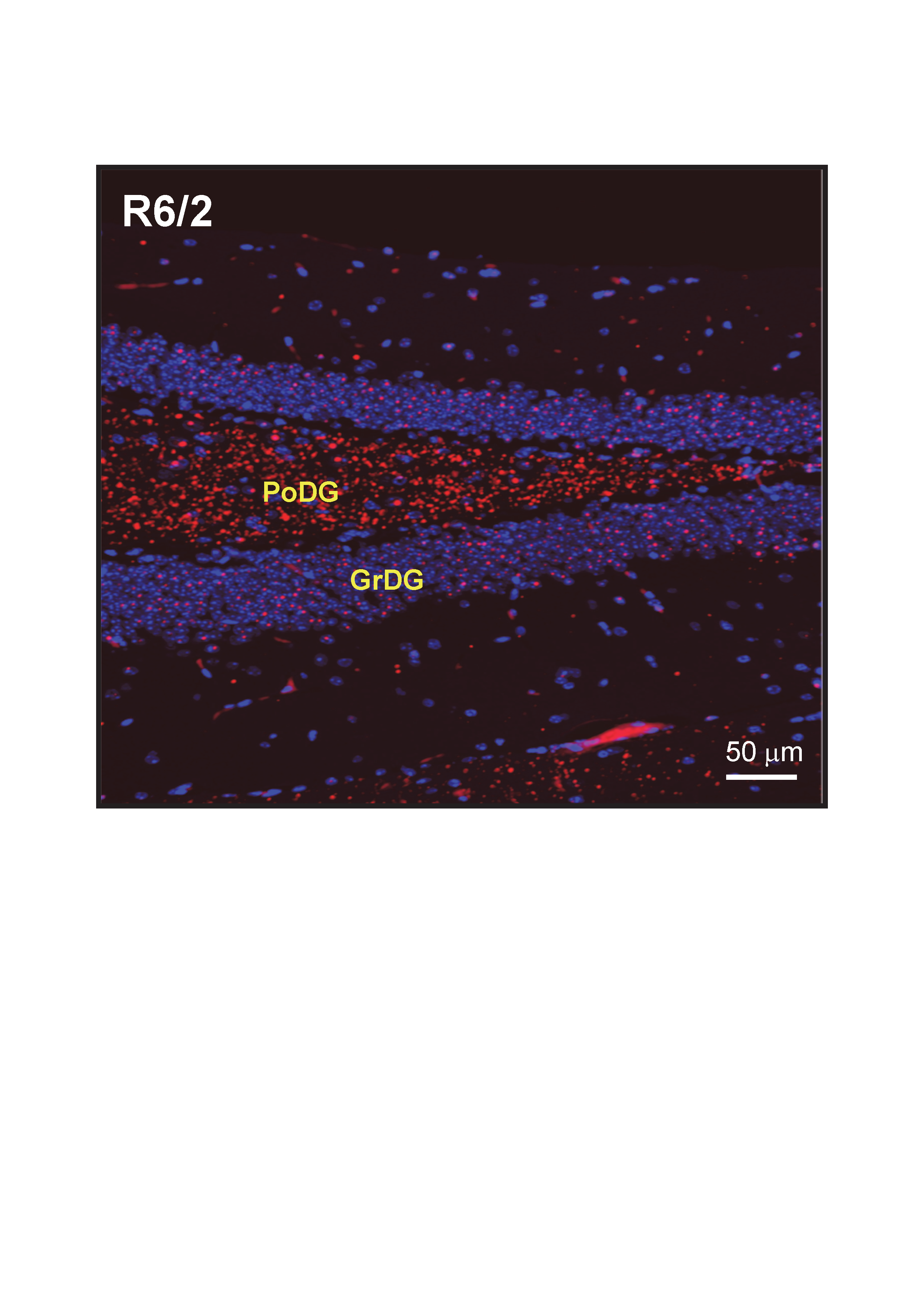

Supplement: Figure S4 — MW8+ mHtt aggregates in the dentate gyrus of R6/2 mice. MW8+ aggregates are shown in the dentate gyrus of a 10-week-old R6/2 mouse to illustrate the high-load of extra-nuclear aggregates in the polymorph layer (PoDG) and the presence of NIIs in neurons of the granule cell layer (GrDG). The image is representative of independent staining experiments using 3 sections/animal and 3 animals/genotype. (TIF) [file pone.0075108.s004.tif]

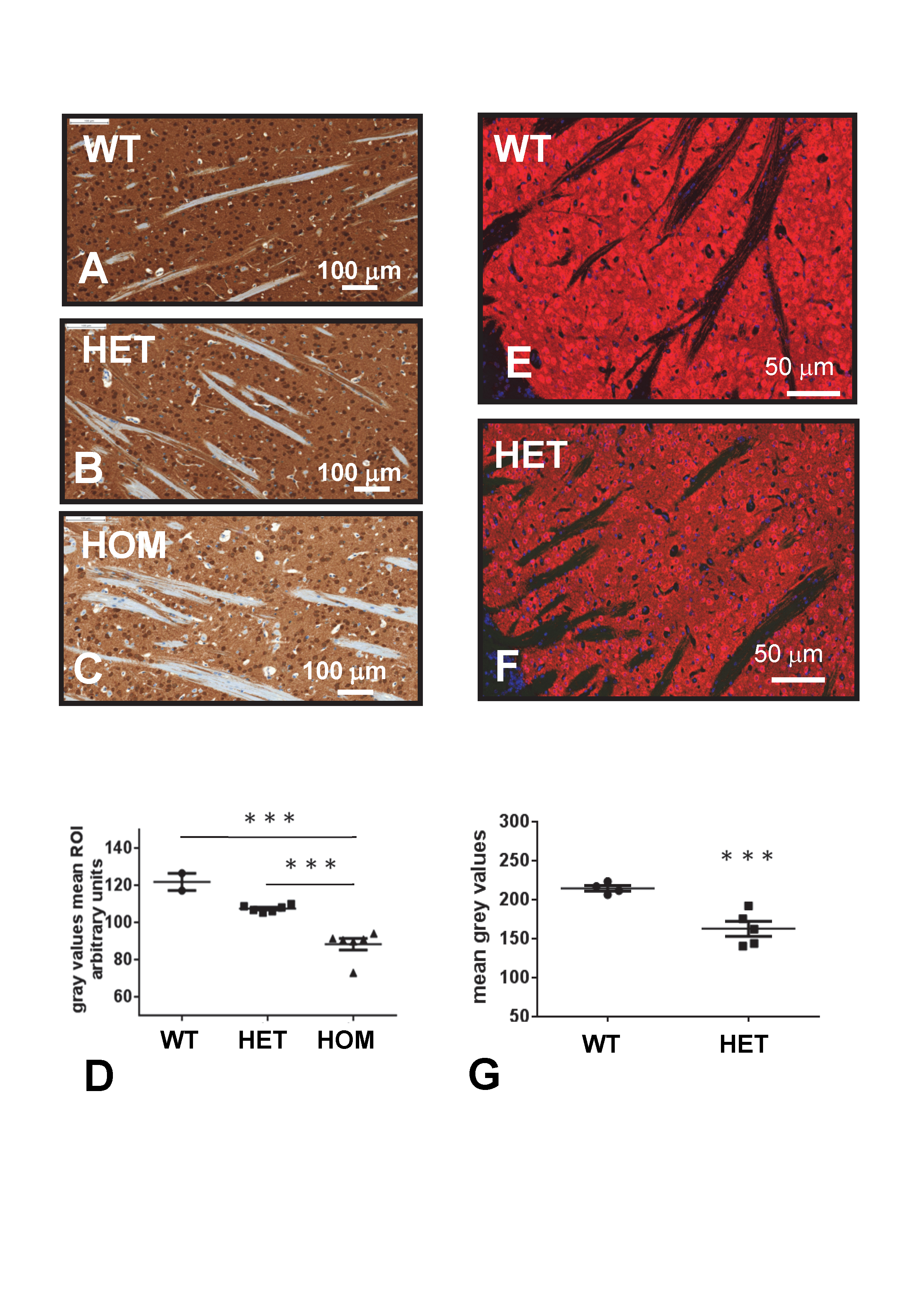

Supplement: Figure S5 — Reduced DARPP32 immunostaining in the HdhQ150 mouse striatum. Images show a comparison of the different DARPP32 immunostaining intensities in the striatum of an 8-month-old wildtype (A), HdhQ150 HET (B) and HdhQ150 HOM mouse (C). (D) Quantitative evaluation of optical density (digital image analysis) revealed statistically significant reductions in DARPP32 staining intensities between HdhQ150 HET (n = 6) and HdhQ150 HOM (n = 6) mice (Mann-Whitney test: p<0.01). (E, F, G) Results are also shown for 10-month-old mice using a different method based on DARPP32 immunofluorescence. Staining intensities in striatum are shown comparing a 10-month-old wildtype (E) and a 10-month-old HdhQ150 HET mouse (F). (G) Digital image analysis of DARPP32 fluorescence intensities revealed significant reduction in DARPP32 signals of HdhQ150 HET (n = 5) as compared to wildtype mice (n = 4). Significance of differences was confirmed using the Mann-Whitney U-test (p<0.01). (TIF) [file pone.0075108.s005.tif]

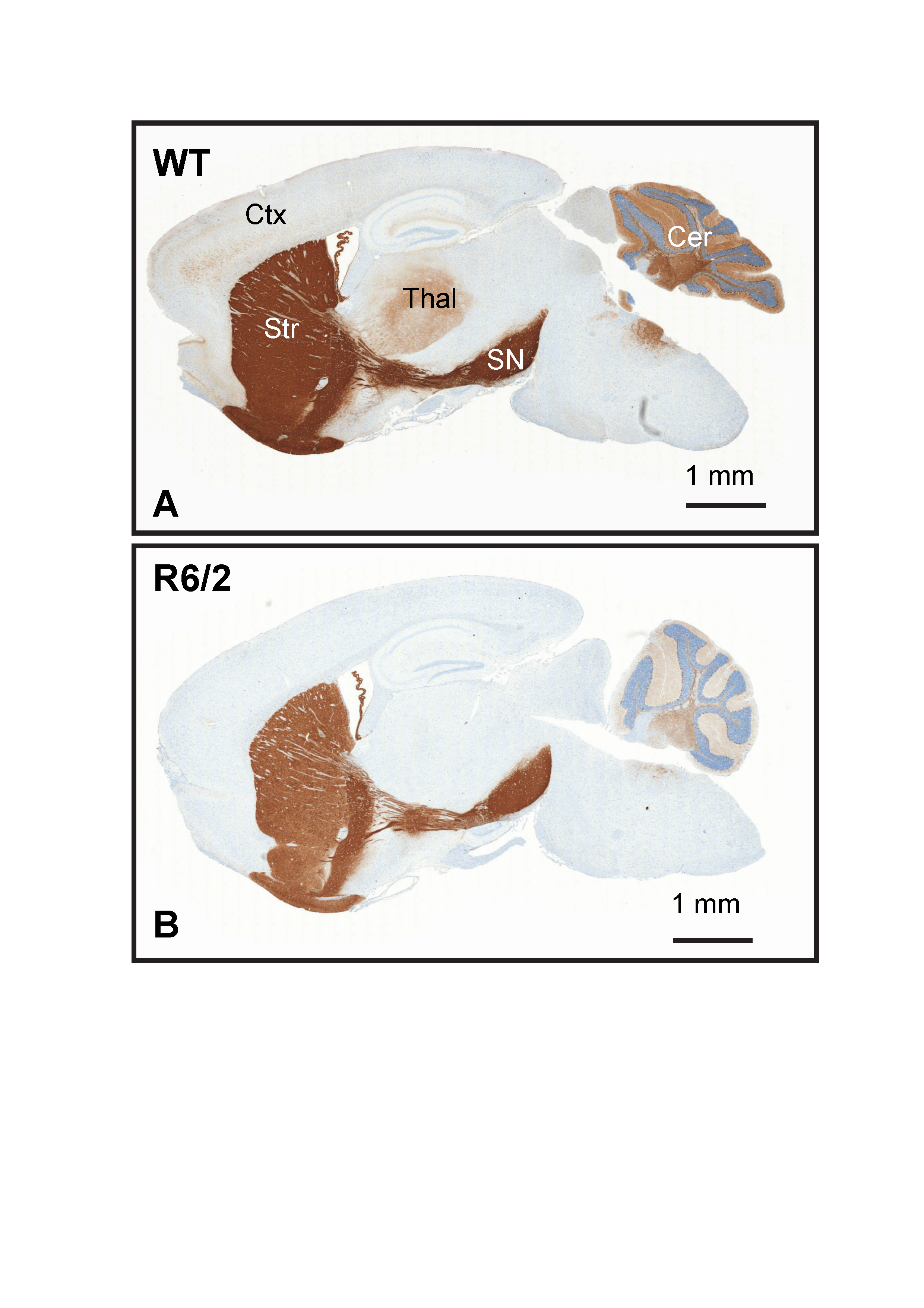

Supplement: Figure S6 — Reduced DARPP32 immunostaining signals in the R6/2 mouse brain. Representative sagittal brain sections are shown of a 10-week-old wildtype (WT) and a 10-week-old R6/2 mouse stained for DARPP32. These show the dramatic reduction of DARPP32 staining signals in the R6/2 striatum (Str), the substantia nigra (SN) and the cerebellum (Cer) and the absence of DARPP32 signals in R6/2 thalamus (Tha) and cortex (Ctx). Images are representative of independent staining experiments using 3 sections/animal, 3 wildtypes and 3 R6/2 mice. (TIF) [file pone.0075108.s006.tif]

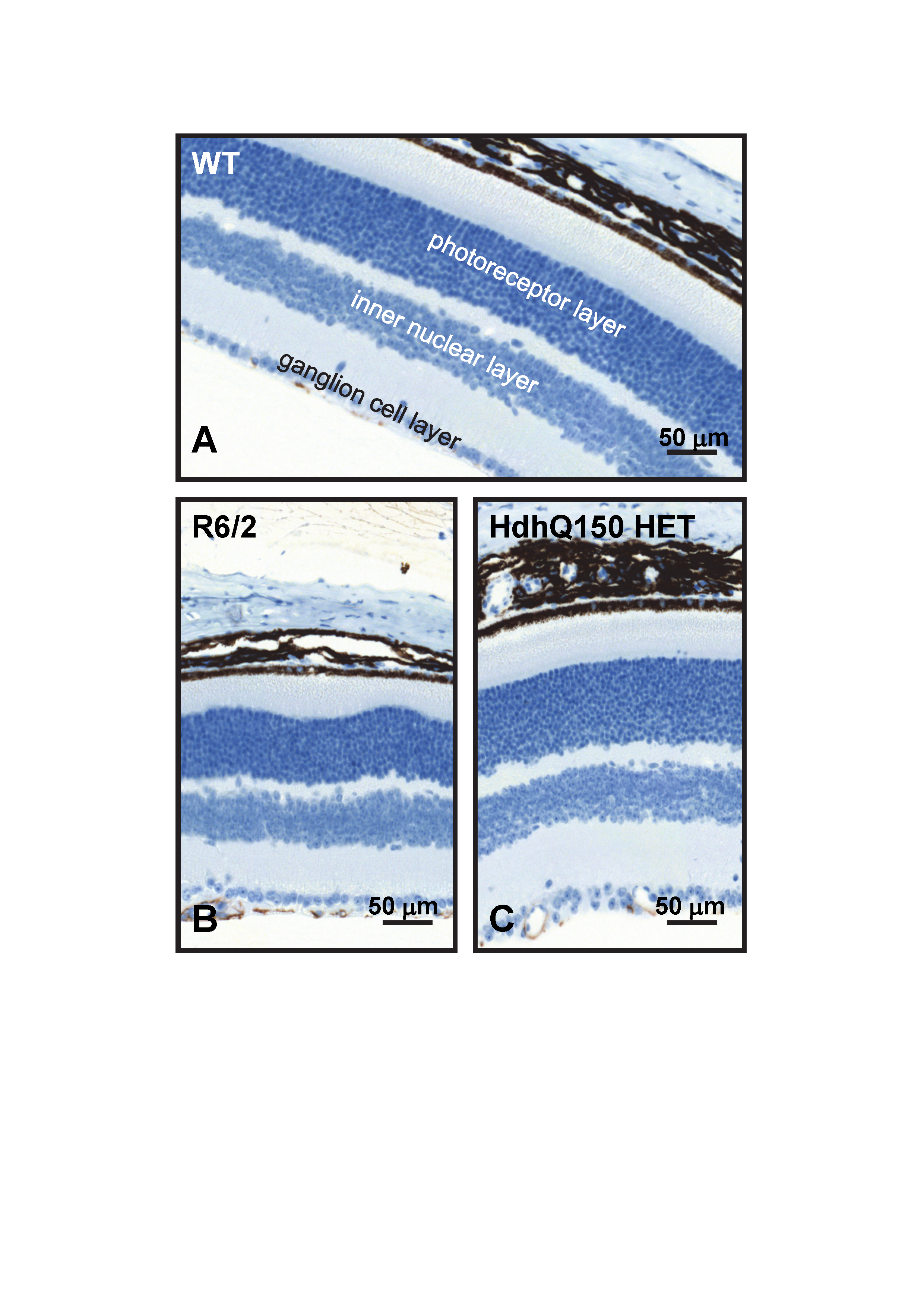

Supplement: Figure S7 — Retina histology of HdhQ150 and R6/2 mice. Images show GFAP staining in Davidson fixed eyes of an 8-month-old wildtype mouse (A), a 10-week-old R6/2 mouse (B), and a 10-month-old HdhQ150 HET mouse (C). None of the retinas displayed enhanced GFAP staining (indicator of Müller glia cell activation). The HdhQ150 HET retina (C) shows no obvious histological abnormalities whereas the photoreceptor layer in the R6/2 retina exhibits a waved structure. The images are representative of independent staining experiments using 3 sections/animal, 3 wildtypes, 4 HdhQ150 HETs and 3 R6/2 mice. (TIF) [file pone.0075108.s007.tif]
